# Elucidating the Oxygen-Activation Mechanism in Nonheme Mn^II^-, Fe^II^-, or Co^II^-Containing MOFs Mimicking Fe^II^/2-Oxoglutarate-Dependent Complexes

**DOI:** 10.1021/acs.inorgchem.6c00307

**Published:** 2026-05-21

**Authors:** Ziyue Huang, Yingqi Li, Xiaotian Zhang, Xi Chen, Jiawei Xu, Haiyan Wei

**Affiliations:** † Jiangsu Key Laboratory of Biofunctional Materials, School of Chemistry and Materials Science, Ministry-of-Education Key Laboratory of Numerical Simulation of Large-Scale Complex Systems, 12534 Nanjing Normal University, Nanjing, Jiangsu 210023, China; ‡ Physical and Theoretical Chemistry Laboratory, Department of Chemistry, 6396 University of Oxford, Oxford OX1 3QZ, U.K.

## Abstract

A Fe/Zn metal–organic framework (MOF) that mimics the Fe^II^/2-oxoglutarate-dependent complex (Fe^II^/2-OGDC) was reported previously, but its oxygen-activation mechanism remains unclear. In the present research, density functional theory (DFT) calculations were used to study this Fe-based system and its Mn- and Co-substituted analogues. We found that all three systems follow the general oxygen-activation chemistry of Fe^II^/2-OGDC but show clear metal-dependent differences. The Fe and Co systems generate high-spin TM^IV^–oxo complexes, whereas the Mn system forms high-spin Mn^III^-oxyl complexes. The energy profiles further show that the formation of the key oxidizing intermediate is more favorable in the Fe and Mn systems than in the Co system. Overall, this work explains how changing the metal center affects oxygen activation in MOF-based enzyme mimics and highlights Mn as a more promising substitute for Fe than Co.

## Graphical abstract

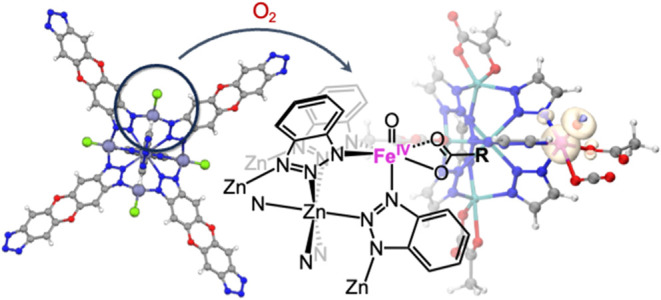

## 1 Introduction

Triplet oxygen (^3^O_2_) is the most accessible oxidant in nature. Although thermochemically favored, the reaction kinetics between ^3^O_2_ and most organic substrates are strongly hindered by spin-forbidden constraints.^1–4^ Most natural solutions highlight the importance of transition metals in biological oxygen activation, where iron is the most common O_2_-binding site. Typical examples include, but are not limited to, the cytochrome P450 (CYP450) family,^5–7^ Fe^II^/2-oxoglutarate-dependent complex (Fe^II^/2-OGDC),^8–10^ Rieske family,^11,12^ etc. Comprehensive reviews have summarized the general catalytic cycle, structural features, and reactivity patterns of Fe^II^/2-OGDCs.^13^

Recent computational studies on nonheme Fe^II^/2-OGDC have provided important mechanistic insights into dioxygen activation, generally supporting the involvement of Fe^III^-superoxo/peroxysuccinate-type intermediates and a high-valent Fe^IV^–oxo oxidant. These studies also suggested that the detailed reactivity can be influenced by the coordination mode of 2-OG, substrate conformation, and structural dynamics.^14–17^ Together with earlier theoretical work on related nonheme iron systems, these studies have established an important mechanistic framework for understanding spin-state changes, O–O bond activation, and the formation of high-valent iron–oxo species.

Much effort has been devoted to extending the catalytic ability of Fe-containing enzymes, especially by mimicking their structure and reactivity in material systems. The CYP450 family uses a planar Fe–N_4_ single-atom site as the O_2_-binding position. Due to the stable, rigid, and graphene-like coordination structure of the Fe-porphyrin cofactor, the Fe–N_4_ single-atom site is well developed and characterized in single-atom catalysts (SAC) embedded in carbon materials, showing similar C–H hydroxylation catalytic capability in the presence of oxygen.^18–20^ However, in other Fe-containing kinases, instead of a stable cofactor structure, Fe (or an iron–sulfur cluster) is coordinated with the solvent and amino acids, forming a flexible coordination structure, awhich makes it difficult to design and reconstruct similar single-atom sites in material systems.^21–24^

It is worth noting that a recent study by Hou et al.^25^ reported the first successful example of building Fe^II^/2-OGDC-like sites in metal–organic framework (MOF) systems, which realized the reactivity of oxygen activation, C–H hydroxylation toward cyclohexane, and selective ethanol oxidation. In biological systems, Fe^II^/2-OGDC utilizes oxygen and 2-oxoglutarate (2-OG; also known as α-ketoglutarate, α-KG) to catalyze redox reactions toward a broad range of substrates, typically through dioxygen activation to generate a high-valent Fe^IV^–oxo oxidant, showing monooxygenase or dehydrogenase reactivity.^26–28^ In the resting state, the Fe^II^/2-OGDC family uses Fe^2+^ as a catalytic center, which is usually coordinated with solvent water and three neighboring histidine (HIS) or aspartate (ASP) residues. The general catalytic mechanism is shown in Figure 1a, where a high-valent Fe^IV^–oxo complex is responsible for C–H activation via the hydrogen atom transfer (HAT) process, followed by hydroxyl rebound to give a hydroxylation product (oxygenase reactivity) or another HAT to give a desaturation product (dehydrogenase reactivity). The MOF systems designed by Hou et al. mimicking a Fe^II^/2-OGDC single-atom site have a slightly different coordination environment with three coordinated 1,2,3-triazole ligands embedded in the frameworks (Figure 1b), where pyruvate (prv) and 3,3-dimethyl-2-oxobutyrate (moba) were used to replace and take over the function of the 2-OG ligand. However, the detailed oxygen-activation mechanism of this MOF system remains unclear, and it is unknown whether analogous Mn- and Co-substituted systems can support similar Fe^II^/2-OGDC reactivity.

**Figure 1:**
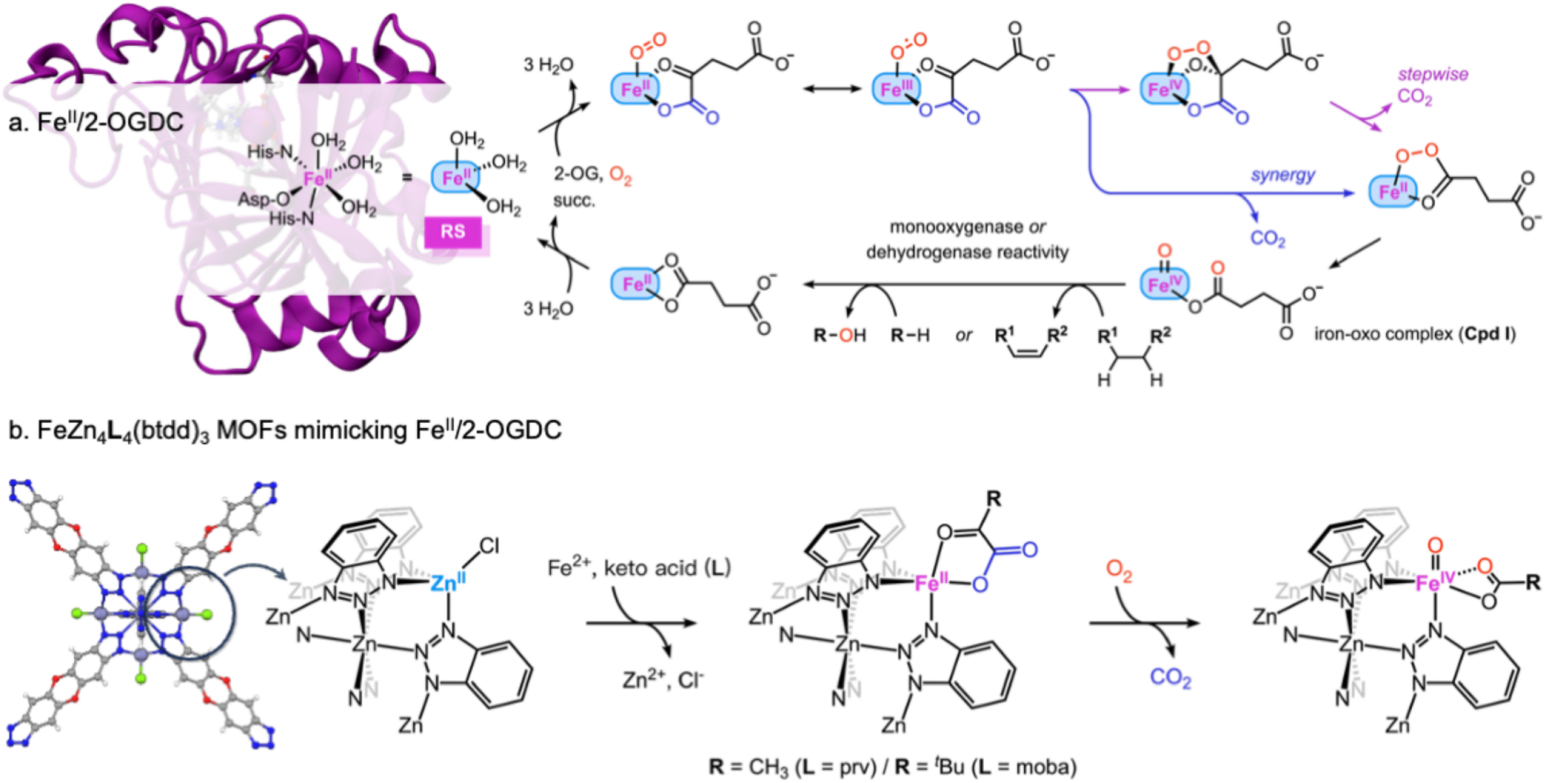
(a) Active site structure and oxygen-activation mechanism of the Fe^II^/2-OGDC family showing monooxygenase and dehydrogenase reactivities. (b) Structure of FeZn_4_ **L**_4_(btdd)_3_ MOFs (H_2_btdd = bis­(1*H*-1,2,3-triazolo [4,5-*b*],[4′,5′-*i*])­dibenzo­[1,4]­dioxin; **L** = prv or moba) mimicking the Fe^II^/2-OGDC family.

Biocatalysis is well-known to have high efficiency and selectivity; however, compared with the material system, it still has some practical drawbacks before massive production and industrial application, such as sensitivity to the environment, high cost, limited substrates, etc. Therefore, a theoretical understanding of the rarely reported material systems mimicking nonheme-type Fe-containing enzymes will be of great importance for future designs. Clarifying these issues is important for establishing metal-dependent design principles for MOF-based mimics of nonheme oxygenases. In this work, our theoretical results reveal how triplet oxygen is utilized by the FeZn_4_(prv)_4_(btdd)_3_ MOF system mimicking the Fe^II^/2-OGDC structure and reactivity.

## 2 Methodology

To simulate the MOF structure, a cluster model of FeZn_4_(prv)_4_(btdd)_3_ was built. All geometric optimizations and frequency calculations were performed under the PBE0-D3­(BJ)/def2-SVP level of theory^29,30^ using the Gaussian16 (revision C.01) program.^31^ PBE0-D3­(BJ) was selected because dispersion-corrected hybrid functionals are widely used in mechanistic studies of transition-metal systems and can provide a reasonable description of localized metal-centered electronic structures.^32^ We note that the absolute spin-state gaps and barriers may depend on the exchange-correlation functional. Therefore, our discussion focuses on qualitative mechanistic trends supported by structural, spin density, UNO/LOBA, and other analyses. The thermal correction to the Gibbs free energy at 298 K (*G*_corr_) was obtained at the same level. The IEF-PCM implicit solvent model was adopted to describe the aqueous environment.^33–35^ The IEF-PCM continuum model cannot explicitly describe pore-confined solvent organization, solvent configurational entropy, or strong local solvent–MOF interactions such as hydrogen bonding. Nevertheless, because all spin states and metal analogues were treated using the same cluster model and solvation protocol, the calculated free energies should be adequate for comparing the qualitative trends across different spin states and metal centers, although the absolute barriers may be affected by explicit solvent effects. To obtain the precise electronic energy (*E*_ele_), single-point calculations were performed based on the optimized structure under the PBE0-D3­(BJ)/def2-TZVP level of theory. Potential energy surface (PES) scans were performed to locate the TS structures using the optimized reactant complex (RC) by scanning the bond coordinates, and then the structures corresponding to the maximum energy points in the scans were optimized using the Berny algorithm.^36^ Optimized TS structures were subjected to intrinsic reaction coordinate (IRC) calculations to confirm that the reactants and products could be connected through the TS while moving in the forward and backward pathways.^37^ The minimum energy crossing point (MECP) was optimized using the branching plane updating algorithm (BPUPD)^38^ with the XMECP code,^39^ with the energy and gradient obtained under the same level of theory as the optimization of the minimum point. Multiwfn (development version 3.8)^40^ was used for wave function analysis, and the Visual Molecular Dynamics (VMD, version 1.9.4)^41^ program was used for visualization. A limited gCP^42,43^ BSSE analysis for representative competing Mn barriers is provided in Table S6, showing that BSSE has a negligible effect on the relative barrier trends relevant to the mechanistic conclusions.

To model the local reaction environment of the MOF active site, a cluster model was constructed by truncating the framework around the metal center of the parent MOF structure. The model retains the complete first coordination sphere of the metal and the chemically relevant nearby framework fragments surrounding the reactive site, including the prv ligand and atoms directly involved in oxygen activation. Truncation was performed at positions remote from the reactive center, and the resulting dangling bonds were saturated with hydrogen atoms. Because the present MOF is an open-framework system, oxygen activation chemistry is expected to be governed primarily by the local coordination environment of the metal center. Although such a cluster model cannot fully describe the long-range framework effects or the full flexibility of the extended MOF lattice, it is appropriate for comparing the Fe, Mn, and Co analogues at the mechanistic level, where the key bond-breaking and -forming events occur locally at the metal site.

## 3 Results and Discussion

### 3.1 Oxygen Activation by FeZn_4_(prv)_4_(btdd)_3_

#### 3.1.1 Iron–Dioxygen Complex

All possible spin-state combinations of Fe^II^ and ^3^O_2_ are listed in Figure S1, which may lead to triplet, quintet, and septet states, noted in Figure 2 as ^3^ **IM1**, ^5^ **IM1**, and ^7^ **IM1**, respectively. Computational results showed that ^7^ **IM1** has a long Fe–O distance of 3.31 Å (Figure 2c), which shows that oxygen is bound to the active site by noncovalent interactions. This weak interaction is also supported by the IGMH analysis,^44^ as shown in Figure S4. The spin density difference (SDD) population numbers showed that Fe^II^ and ^3^O_2_ have parallel populations of 3.86 and 2.00 in ^7^ **IM1**, where the electron exchange interaction enhanced its stability to the lowest spin state. Therefore, ^7^ **IM1** was chosen as the zero-energy level for further calculations. Nevertheless, the spin-parallel states of Fe^II^ and ^3^O_2_ are not reactive states for further single-electron transfer (SET) because all unpaired electrons are parallel to each other, as pointed out by previous calculations on the Fe^II^/2-OGDC family.^45–47^

**Figure 2:**
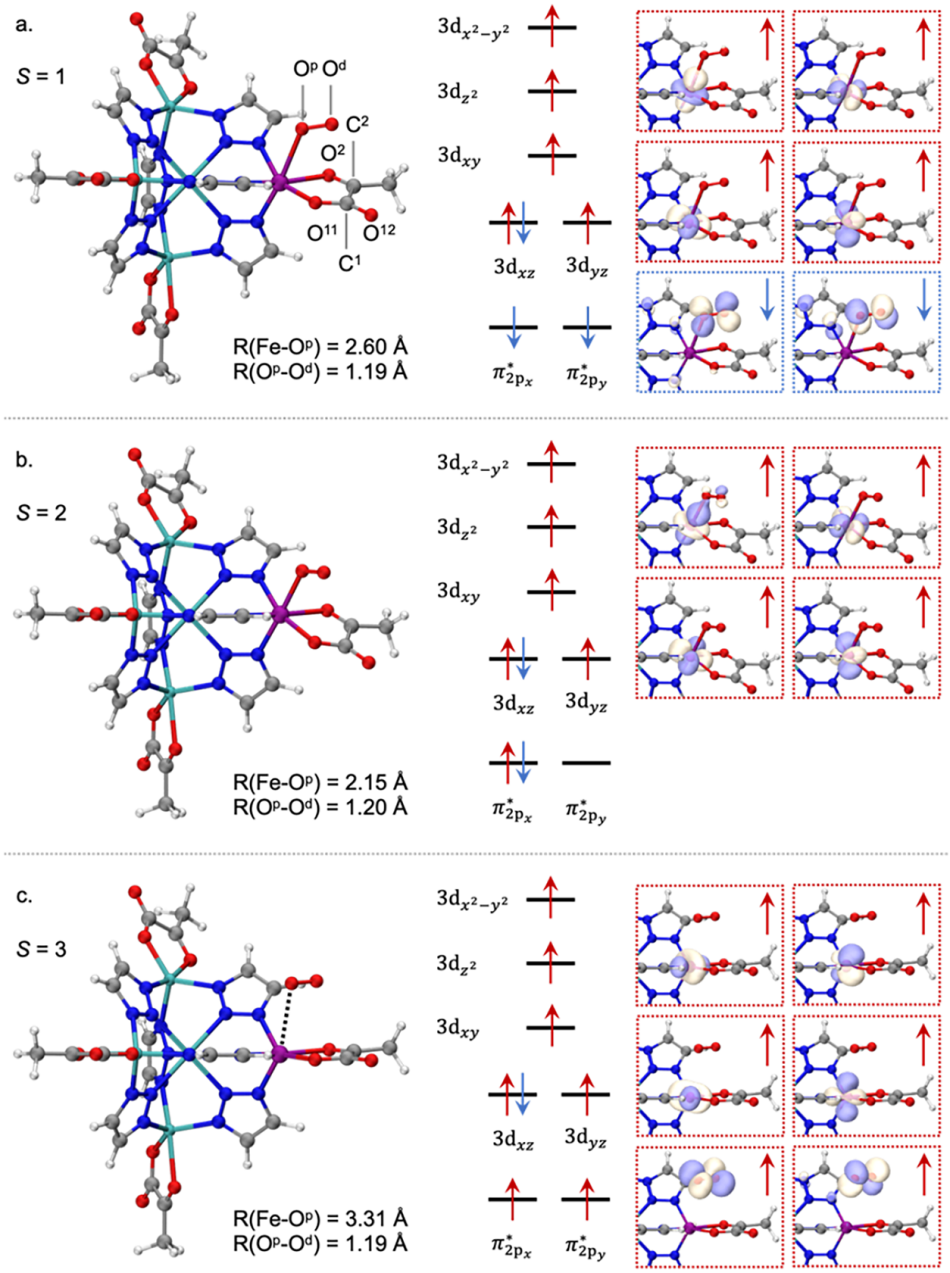
Calculated structure, electron configuration, and singly occupied orbitals of possible spin states of iron–oxygen complexes formed in the active site of FeZn_4_(prv)_4_(btdd)_3_. (a) ^**3**^ **IM1** (*S* = 1, 2.3 kcal·mol^–1^), (b) ^**5**^ **IM1** (*S* = 2, 12.9 kcal·mol^–1^), and (c) ^**7**^ **IM1** (*S* = 3, 0.0 kcal·mol^–1^).

For other possible spin-states, ^3^ **IM1** and ^5^ **IM1** are 2.3 and 12.9 kcal·mol^–1^ higher than ^7^ **IM1**. Both ^3^ **IM1** and ^5^ **IM1** adopt an end-on coordination configuration (Figure 2b,c), where the Fe–O distance is reduced to 2.60 and 2.15 Å in ^3^ **IM1** and ^5^ **IM1**, respectively, indicating the formation of a σ_Fe–O_ bond upon adsorption. Compared with noncovalent adsorption (1.19 Å), the O–O distance does not change much during covalent adsorption (1.19 Å in ^3^ **IM1** and 1.20 Å in ^5^ **IM1**). This indicates that SET has not been completed in any spin state of **IM1**, as the O–O bond retains its feature in the free state, giving the formal oxidation state of the Fe^II^–O_2_ complex. The spin-state ^3^ **IM1** is thermochemically more favored compared to that of ^5^ **IM1**. As can be seen from the SDD population numbers (Figure 2b), the antiferromagnetic coupling caused by opposite SDD populations on the Fe^II^ center (+3.85 e) and oxygen (−0.96 and −1.01 e) in ^3^ **IM1** contributes to its stability.

Possible alternative oxygen-activation pathways were also considered. In addition to the explicitly calculated spin states and coupling alternatives, several formally possible routes were excluded on chemical grounds. A side-on η^2^-O_2_ binding mode would require two adjacent open coordination sites or substantial disruption of the prv/triazole coordination environment. An O^p^ attack on the α-keto C^2^ atom would disrupt the M–O interaction, whereas an O^d^ attack would preserve the metal–dioxygen connection and provide a direct route for coupled C–O bond formation, electron transfer, and decarboxylation. The attack at the carboxylate C^1^ atom is also unlikely because it does not naturally lead to C^1^–C^2^ oxidative cleavage or TM–oxo/oxyl formation. Finally, direct O–O cleavage before prv participation was not considered chemically viable because the formation of a high-valent TM–oxo/oxyl species requires electron supply from 2-oxoacid oxidative decarboxylation. Therefore, the present pathway search focuses on the chemically relevant O^d^ attack/decarboxylation mechanism across accessible spin-state surfaces.

#### 3.1.2 Multispin-State Oxygen Activation

For clarity, O^p^ denotes the oxygen atom coordinated to the metal center, while O^d^ denotes the distal oxygen involved in the attack on the C^2^ atom of prv. The labels C^1^ and C^2^ are shown in Figure 2a. Despite the noncovalent oxygen adsorption structure (^7^ **IM1**_Fe_), ^3^ **IM1**_Fe_ is the most favored initial adsorption structure for oxygen activation. Two spin states were located for the triplet potential energy surfaces, as marked with suffixes **A** and **B** in Figure 3a. Pathway **A** indicates that the unpaired electrons on the Fe and O_2_ parts have opposite spins to form antiferromagnetic coupling, while pathway **B** indicates no antiferromagnetic coupling. This was confirmed by the SDD population numbers (Table S1).

**Figure 3:**
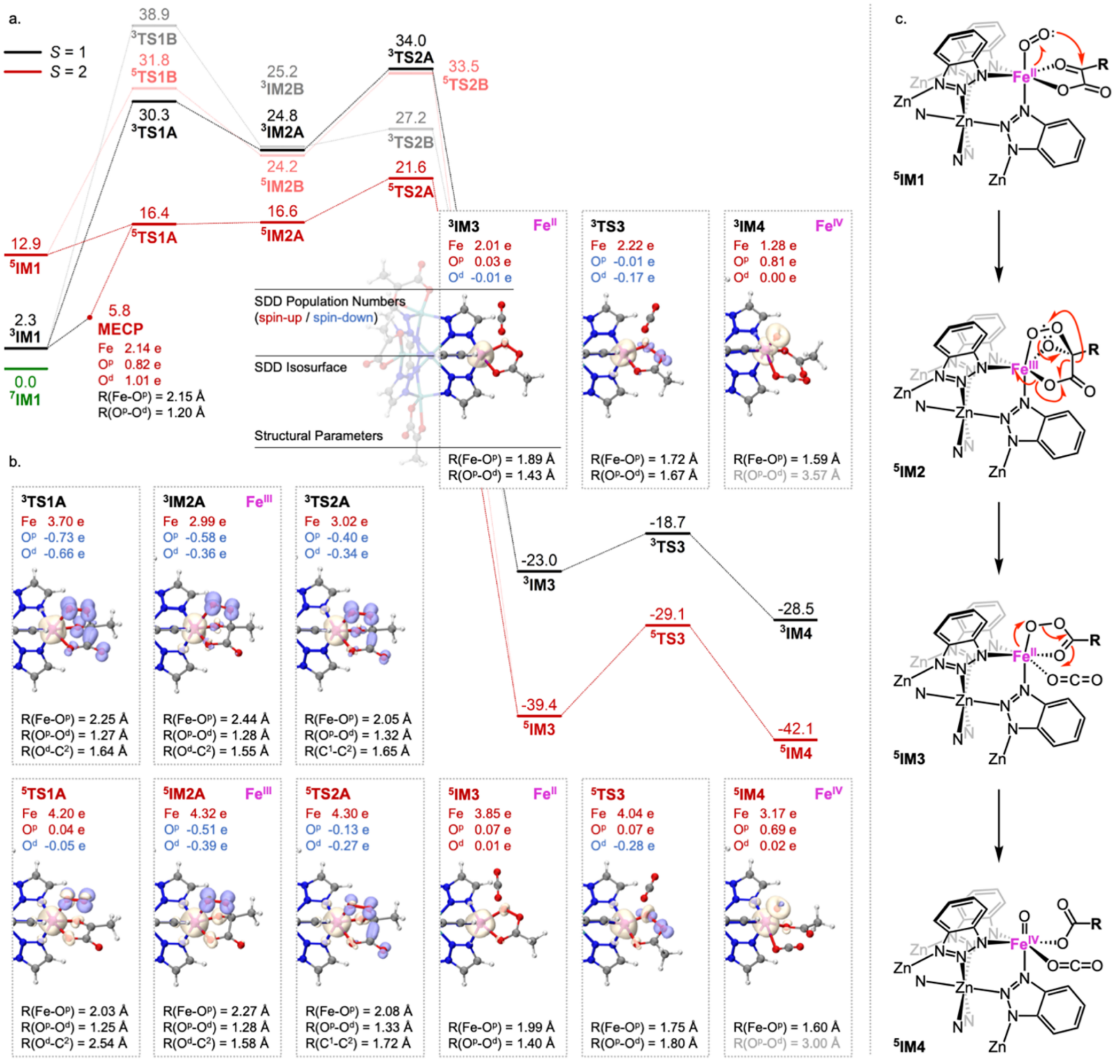
(a) Multispin-state Gibbs free energy diagram of oxygen activation by FeZn_4_(prv)_4_(btdd)_3_ (energy in kcal·mol^–1^ relative to ^7^ **IM1**_Fe_). (b) Structure of the active site and spin density difference. (c) Electron transfer route of the favored reaction pathway.

As revealed by the potential energy surface (Figure 3a), on the triplet surface, the Gibbs free energy barriers for the distal oxygen (O^d^) attacking the C^2^ atom of the prv ligand are 28.0 kcal·mol^–1^ (^3^ **TS1A**_Fe_) and 36.6 kcal·mol^–1^ (^3^ **TS1B**_Fe_) relative to ^**3**^ **IM1**_Fe_. The antiferromagnetic coupling pathway has a lower barrier than the pathway without antiferromagnetic coupling, although the barrier is still too high to be overcome at room temperature, and the following transition states (^3^ **TS2A**/**B**_Fe_ and ^3^ **TS3**) have lower barriers. Therefore, triplet states are not the desired spin states for the oxygen-activation process. This is consistent with the conclusions reached in biochemical systems. Considering that the final Fe^IV^–oxo complex adopts a quintet spin state, spin crossing is crucial to the whole pathway, and the minimum energy crossing point (MECP) should be located before reaching **TS1**_Fe_ to lead the spin-state transition from ^3^ **IM1**_Fe_ to ^5^ **TS1A**/**B**_Fe_. As shown in Figure 3a, this MECP is 3.5 kcal·mol^–1^ higher than ^3^ **IM1**_Fe_, which is available with only thermal activation at room temperature. On the quintet potential energy surfaces, the Gibbs free energy barriers of ^5^ **TS1A**_Fe_ and ^5^ **TS1B**_Fe_ are 3.5 kcal·mol^–1^ and 18.9 kcal·mol^–1^, respectively, showing that both quintet spin states are available at room temperature, and pathway **A** with antiferromagnetic coupling is more favored. The productive Fe pathway in the present MOF model is somewhat higher in energy than those reported for several nonheme Fe enzyme systems.^14^ However, this increase is still moderate and remains within a chemically accessible range. This is reasonable because the present system is a more robust artificial MOF mimic, whereas enzyme active sites are more finely tuned by the surrounding protein environment.

As shown in Figure 3b, in the pathway ^3^ **A**, the rate-determining transition state is closer to its product intermediates (the one next to this transition state), ^3^ **IM2A**_Fe_, in terms of structural parameters. Specifically, in ^3^ **TS1A**_Fe_, the key reaction coordinate *R*(O^d^–C^2^) is 1.64 Å, which is close to 1.55 Å in its product intermediate ^3^ **IM2A**_Fe_, showing that the O^d^–C^2^ bond has almost been formed at the transition state, while *R*(O^d^–C^2^) = 3.22 Å shows no interaction in reactant intermediate ^3^ **IM1**. Besides, the Fe–O^p^ coordination bond is strengthened during this step, as ^3^ **TS1A**_Fe_ has a shorter *R*(Fe–O^p^) = 2.25 Å compared with 2.60 Å in ^3^ **IM1**_Fe_, which is closer to 2.44 Å in ^3^ **IM2A**_Fe_. *R*(O^p^–O^d^) is the same case, with O^p^–O^d^ being elongated from 1.19 Å in ^3^ **IM1**_Fe_ to 1.27 Å in ^3^ **TS1A**_Fe_, and finally, reaching 1.28 Å in ^3^ **IM2A**_Fe_. The progressive O–O activation is also supported by the Mayer Bond Order (MBO) analysis.^48^ The O^p^–O^d^ MBO decreases from 1.13 in ^3^TS1A_Fe_ to 0.57 in ^3^TS3_Fe_, whereas the Fe–O^p^ bond order increases from 0.25 to 1.03, consistent with O–O bond weakening coupled with the Fe–O bond formation (Table S4). All these structural parameters indicate that ^3^ **TS1A**_Fe_ resembles the structure of product intermediate ^3^ **IM2A**_Fe_, which, according to the Hammond’s postulate,^49,50^ is a “late transition state” and corresponds to a highly endergonic reaction. Nevertheless, compared with the “early transition state” ^5^ **TS1A**_Fe_, which resembles its reactant intermediate ^5^ **IM1**_Fe_, ^3^ **TS1A**_Fe_ gives a much higher barrier of 28.0 kcal·mol^–1^ than ^5^ **TS1A**_Fe_ does (3.5 kcal·mol^–1^). As for ^3^ **TS2A**_Fe_ and ^5^ **TS2A**_Fe_, both transition states resemble their reactant intermediates (^3^ **IM2A**_Fe_ and ^5^ **IM2A**_Fe_), which explains why ^3^ **TS2A**_Fe_ and ^5^ **TS2A**_Fe_ have similar low Gibbs free energy barriers of 9.2 and 5.0 kcal·mol^–1^, respectively.

We may naturally ask why ^5^ **TS1A**_Fe_ resembles ^5^ **IM1**_Fe_, and why ^3^ **TS1A**_Fe_ does not. The electronic structure provides an answer to this question. As shown in Figure 2b, the less-favored spin state of ^5^ **IM1**_Fe_ can be described as high-spin Fe^II^ and low-spin close-shell ^1^O_2_, instead of ^3^O_2_ in ^3^ **IM1**_Fe_ or ^7^ **IM1**_Fe_, which is the reason for its high energy level. ^5^ **TS1A**_Fe_ resembles ^5^ **IM1**_Fe_ in structure but not in electronic structure. As shown in Figure 3b, although the SDD population shows almost no net spin density difference on both oxygen atoms in ^5^ **TS1A**_Fe_, the isosurface still presents a notable distribution of both spin-up (shown in white) and spin-down (shown in blue) densities, showing the shape of a pair of orthogonal π* orbitals and indicating the electronic state of open-shell ^1^O_2_. The pathway from ^3^ **IM1**_Fe_ to ^5^ **TS1A**_Fe_ via MECP can therefore be concluded as intersystem crossing from the ^3^O_2_ state to the open-shell ^1^O_2_ state with one unpaired electron flipped. The structural variation on the ^5^ **A** potential energy surface, including ^3^ **IM1**_Fe_ and MECP, is accompanied by electronic structure relaxation, resulting in a slow energy increase. However, in the ^3^ **A** potential energy surface, although ^3^ **TS1A**_Fe_ highly resembles the structure of ^3^ **IM2A**_Fe_, the electronic structure relaxation is notably delayed compared with the geometry. The SDD population number of Fe atoms merely changes from 3.84 e in ^3^ **IM1**_Fe_ to 3.70 e in ^3^ **TS1A**_Fe_, which is far from the targeted 2.99 e in ^3^ **IM2A**_Fe_, which is a medium-spin Fe^III^. Distorted geometry, but without a fully relaxed electronic structure, increases the energy significantly, leading to a high Gibbs free energy barrier of ^3^ **TS1A**_Fe_.

Another important discovery in ^3^ **TS1A**_Fe_ and ^5^ **TS1A**_Fe_ is revealed by the electronic structure. Generally, the process of O^d^-attacking the C^2^ atom can be divided into two parts: (1) addition of the C^2^ atom to form a C–O bond and (2) electron transfer from Fe^II^ to the π* of O_2_. This feature can be classified as bond formation coupled electron transfer (BFCET), and it is the same for both ^3^ **TS1A**_Fe_ and ^5^ **TS1A**_Fe_. Both localized molecular orbitals and LOBA analysis (Table S3) show the feature of Fe^III^ in ^3^ **IM2A**_Fe_ and ^5^ **IM2A**_Fe_. The difference between ^3^ **TS1A**_Fe_ and ^5^ **TS1A**_Fe_ is that, in ^3^ **TS1A**_Fe_, the bond formation is already complete because its structure resembles that of ^3^ **IM2A**_Fe_, while the electron has not yet been transferred. This should be further noted as an asynchronous BFCET, where the electron transfer is triggered by bond formation. As shown in Figure 3b, the SDD isosurface of ^3^ **TS1A**_Fe_ has a notable distribution on the σ-framework of the prv ligand, especially including the C^1^–C^2^ σ orbital. This indicates that in ^3^ **TS1A**_Fe_, the C^1^–C^2^ σ bond has a radical feature, although it is reserved in ^3^ **IM2A**_Fe_. Bond formation in ^3^ **TS1A**_Fe_ is therefore a radical attack process, in which the C^1^–C^2^ σ bond acts as an electron transfer bridge. This can be described as shown in Figure S2a using the arrow-pushing scheme, where the red arrows show the radical attack and the blue arrows show the electron transfer route from Fe^II^ to the prv ligand. However, the opposite is true for ^5^ **TS1A**_Fe_, where electron transfer has already been completed, resulting in a high-spin Fe^III^ in the transition state, while bond formation has yet to occur, as indicated by the long *R*(O^d^–C^2^) distance of 2.54 Å. The SDD distribution is not observed on the prv ligand, and electron transfer only occurs between Fe^II^ and O_2_. The C–O bond formation should be characterized as nucleophilic addition, as indicated by the arrow-pushing scheme in Figure S2b, discarding the possibility of a radical attack. Thus, ^5^ **TS1A**_Fe_ should also be noted as an asynchronous BFCET, but nucleophilic addition is triggered by electron transfer, which activates ^3^O_2_ to be in the superoxide form with higher nucleophilicity.

After generating **IM2A**_Fe_, the ^3^ **A** and ^5^ **A** pathways share a similar electron transfer route in the following reactions. The Fe atom adopts a medium spin state in the ^3^ **A** pathway and a high-spin state in the ^5^ **A** pathway, with an energy gap of 8.2∼16.4 kcal·mol^–1^ between the spin states, indicating that the two spin states are well separated and both pathways are spin-conservative. This also holds true for ^3^ **B** and ^5^ **B**. The Fe atom adopts a low-spin state in the ^3^ **B** pathway and a medium-spin state in the ^5^ **B** pathway, as shown in Table S1. Fe^III^ in **IM2A**_Fe_ is first reduced to Fe^II^ in **IM3A**_Fe_, as verified by LOBA analysis (Table S3). The electronic state of **TS2A**_Fe_ should be described as radical decarboxylation, as the SDD distribution is observed on the C^1^–C^2^ σ orbital, regardless of the different spin states (Figure 3b). Electron transfer is triggered by the superoxide radical, which is reduced to peroxide by receiving one electron from the C^1^–C^2^ σ orbital and leads to the heterolysis of the C^1^–C^2^ σ bond. This makes the carboxyl group require one more electron to fulfill the structure of CO_2_, which is provided by the heterolysis of the Fe^III^–O^11^ bond, and Fe^III^ is reduced to Fe^II^ at the same time. The electron transfer route is shown in Figure 3c. If we consider Fe^III^ and superoxide motifs as a whole, this transition state can be considered a generalized reductive elimination model that adopts a radical mechanism.

The generation of ^5^ **IM4**_Fe_ via ^5^ **TS3**_Fe_ has a Gibbs free energy barrier of 10.3 kcal·mol^–1^, which can be easily overcome at room temperature. The SDD isosurface shows no radical feature in ^5^ **TS3**_Fe_, which confirms that the transition state is a 2-electron transfer, converting Fe^II^ to Fe^IV^ along with the cleavage of the peroxide bond. The final reactive oxygen species (ROS) **IM4**_Fe_, also known as **Cpd I**_Fe_ in biochemical references, has a quintet ground state, which is 13.6 kcal·mol^–1^ lower than the triplet state. Here, we also added the “Fe” suffix to distinguish it from the Mn- and Co-containing analogues. This result is identical to that observed for nonheme Fe-containing enzymes. The SDD isosurface of ^5^ **IM4**_Fe_ presents an antibonding feature with a node plane between the Fe and O atoms, as well as σ-symmetry, which corresponds to the combination of two Fe–O π* orbitals (π_xz_^*^ and π_yz_^*^). The Fe atom has a 3.17 SDD population number, which is close to 3 unpaired α electrons, one of which can be attributed to the π* orbitals, while the other two unpaired electrons are located on the 3d orbitals of Fe^IV^, holding a high-spin d^4^-configuration. This electronic state is similar to that reported for the nonheme Fe^IV^–oxo complex.

The Fe^IV^–oxo bond length is a key factor to reflect the reactivity of **Cpd I**_Fe_. Reported experimental ^3^Fe^IV^–oxo bond lengths lie within 1.60∼1.67 Å, while nonheme ^5^Fe^IV^–oxo has a more flexible coordinating environment and may extend this range to 1.58∼1.68 Å.^51^ As observed by in situ DRIFTS analysis of FeZn_4_(prv)_4_(btdd)_3_, the absorption of ^5^Fe^IV^–oxo bond stretching appears at 831 cm^–1^.^25^ In recently reported bond length data collected by multiple spectroscopic techniques, the synthesized **Cpd I**_Fe_ model with a 1.634 Å bond length gives a Raman signal at 811 cm^–1^,^52^ while the Cpd I_Fe_ model with a 1.65 Å bond length gives an NRVS signal at 809 cm^–1^.^53^ This suggests the Fe^IV^–oxo bond length in FeZn_4_(prv)_4_(btdd)_3_ should be shorter, which is consistent with the 1.60 Å obtained by our DFT calculations.

### 3.2 Oxygen Activation by TMZn_4_(prv)_4_(btdd)_3_ (TM = Mn or Co)

Transition metals, including Mn and Cu, which have flexible oxidation states and more unpaired electrons in the 3d orbitals, may have unique capabilities toward various redox reactions and are commonly involved in nonheme types of metalloenzymes as redox centers. For oxygen activation, Mn, Fe, and Cu are popular candidates among first-row transition metals. However, Cu-catalyzed oxygen activation usually requires multinuclear sites, which are difficult to design and reconstruct in synthesized catalysts. Therefore, biomimetic catalysts mainly focus on Mn- and Fe-containing mononuclear active sites. With one less electron but a similar ionic radius, replacing the Fe^II^ center with Mn^II^ should have little influence on the MOF structure; however, the reactivity of MnZn_4_(prv)_4_(btdd)_3_ is unclear. Although Co^II^-containing enzymes are rarely reported to catalyze oxygen activation, we also included CoZn_4_(prv)_4_(btdd)_3_ to investigate the reactivity with one more electron in the 3d orbitals. Therefore, in the following sections, we present the DFT results of the oxygen-activation process catalyzed by Mn and Co-replaced MOFs, to compare them with the FeZn_4_(prv)_4_(btdd)_3_ MOFs.

#### 3.2.1 MnZn_4_(prv)_4_(btdd)_3_

Replacing Fe^II^ with Mn^II^ in the resting state gives MnZn_4_(prv)_4_(btdd)_3_. As shown in Figure 4a, the final ROS adopts a quartet ground state (^4^ **IM4**_Mn_), which is 11.0 kcal·mol^–1^ lower in Gibbs free energy than its sextet state (^6^ **IM4**_Mn_) and 39.1 kcal/mol lower than its doublet state (^2^ **IM4**_Mn_). From the doublet state, the pathway was excluded from consideration due to the prohibitively high energies of both intermediates (>29 kcal·mol^–1^) and transition states (>40 kcal·mol^–1^). Considering that the antiferromagnetically coupled combination of the high-spin d^5^ configuration of Mn^II^ and ^3^O_2_ also has a quartet ground state, the oxygen activation by MnZn_4_(prv)_4_(btdd)_3_ has one spin-conservative pathway on the quartet potential energy surface, as presented by the black line in Figure 4a.

**Figure 4:**
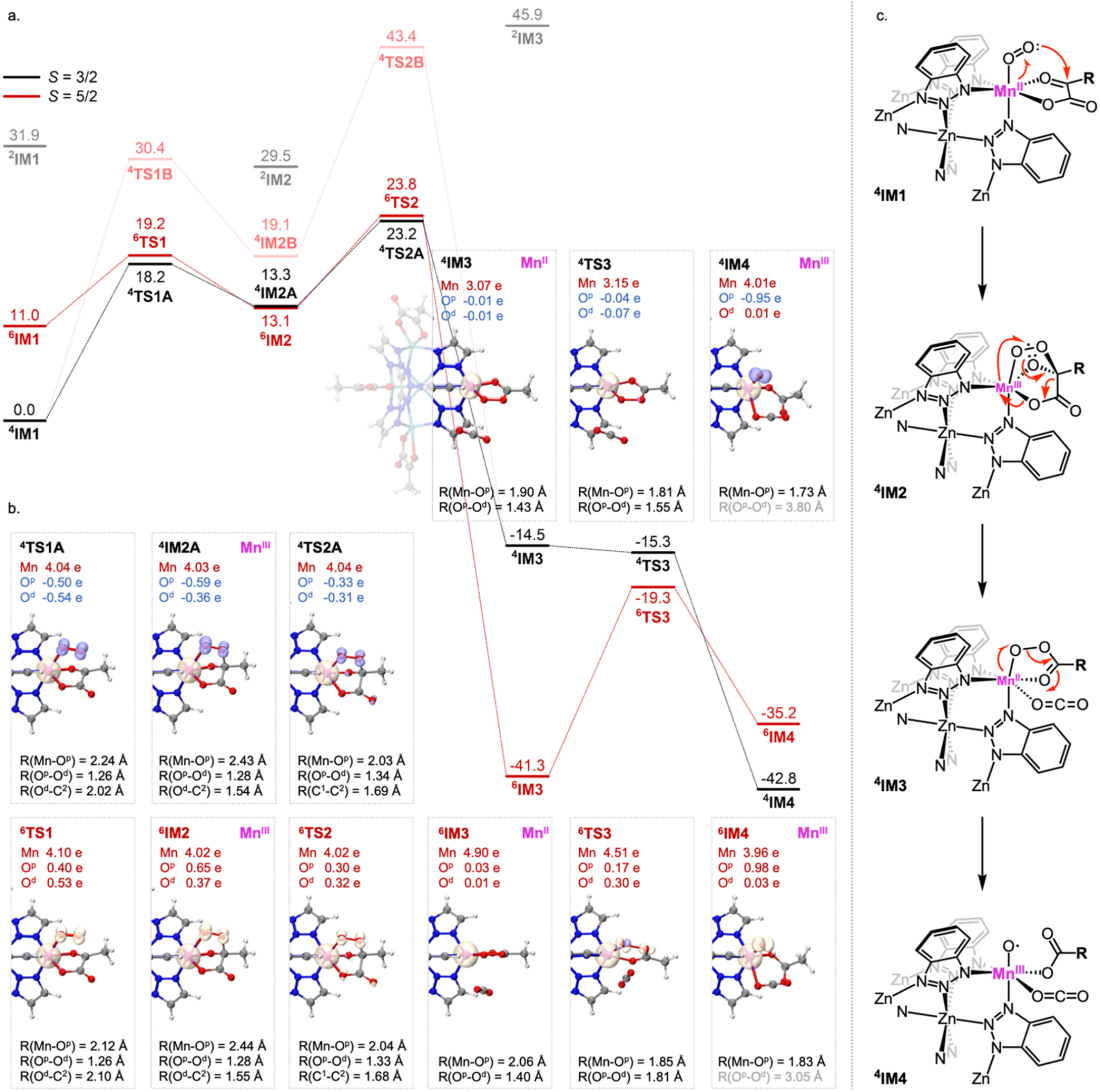
(a) Multispin-state Gibbs free energy diagram of oxygen activation by MnZn_4_(prv)_4_(btdd)_3_ (energy in kcal·mol^–1^ relative to ^4^ **IM1**). (b) Structure of the active site and spin density difference. (c) Electron transfer route of the favored reaction pathway.

Similar to what has been discussed in Fe-catalyzed oxygen activation, the Mn-catalyzed ^4^ **A** pathway (high-spin Mn, with antiferromagnetic coupling) has a much lower energy compared with the ^4^ **B** pathway (medium-spin Mn, without antiferromagnetic coupling). Besides, the sextet potential energy surface provides a lower pathway involving high-spin Mn without antiferromagnetic coupling. Generally, the ^4^ **A** pathway has the lowest Gibbs free energy barrier of 18.2 kcal·mol^–1^ at ^4^ **TS1A**_Mn_ and is the most favored reaction pathway. Sharing the same high-spin Mn center but without antiferromagnetic coupling, the sextet pathway has a close but higher energy compared to the quartet state, and the rate-determining step is ^6^ **TS3**_Mn_, with an energy barrier of 22.0 kcal·mol^–1^. Specifically, in pathways ^4^ **A and** ^**4**^ **B**, the Gibbs free energy barriers for the distal oxygen (O^d^) attacking the C^2^ atom of the prv ligand reach 18.2 kcal·mol^–1^ (^4^ **TS1A**_Mn_) and 30.4 kcal·mol^–1^ (^4^ **TS1B**_Mn_). The first step is the rate-limiting step. Structurally, the O^d^–C^2^ bond length in the quartet transition-state ^4^ **TS1A**_Mn_ shortens from 3.15 Å (in ^4^ **IM1**_Mn_) to 2.05 Å, and finally forms a covalent bond (1.54 Å) to generate the product intermediate ^4^ **IM2A**_Mn_. During this step, the Mn–Op coordination bond is strengthened, which is similar to the case of the Fe-catalyzed system. The difference is that compared with ^5^ **TS1A**_Fe_, ^4^ **TS1A**_Mn_ has a longer Mn–O^p^ bond (^4^ **TS1A**_Mn_: 2.24 Å; ^5^ **TS1A**_Fe_: 2.03 Å), indicating that the Mn–O^p^ coordination bond is weaker than the Fe–O^p^ coordination bond. The following is the C^1^–C^2^ bond cleavage: C^1^–C^2^ bond dissociation in ^4^ **IM2A**_Mn_ (via ^4^ **TS2A**_Mn_ to ^4^ **IM3**_Mn_) has a low barrier (10 kcal·mol^–1^); in contrast, cleavage in ^4^B (via ^4^ **TS2B**_Mn_ to ^4^ **IM3**_Mn_) features a higher barrier (24.3 kcal·mol^–1^) and exergonicity (33.6 kcal·mol^–1^). As shown in Figure 4b, the structural parameters correspond to the elongation and cleavage of the C^1^–C^2^ and O^p^–O^d^ bonds.

The SDD isosurface reveals an important difference between the Mn- and Fe-catalyzed pathways. In the two displayed Fe-catalyzed pathways (Figure 3b), the triplet and quintet electronic states differ at the spin state of the Fe-center; therefore, the medium-spin Fe (triplet state) is highly unfavorable in terms of energy, i.e., the triplet and quintet states are separated by a notable energy gap. In such a case, intersystem crossing from the initial triplet state to the productive quintet state is necessary for oxygen activation, and also methods that may enhance intersystem crossing can be applied to Fe-catalyzed systems to help increase their reactivity. In Mn-catalyzed pathways, especially for **TS1**, **IM2**, and **TS2**, the quartet and sextet states are close in energy because both electronic states have the same high-spin Mn and only differ with or without antiferromagnetic coupling between the Mn center and ligand, as shown in Figure 4b. In most mononuclear metallic complexes, antiferromagnetic coupling only slightly decreases the energy. However, intersystem crossing between these two states is not favored due to the electron spin flip without a change in the orbital angular momentum, as pointed out by El-Sayed’s rule. Thus, intersystem crossing and MECP are not involved in Mn-catalyzed pathways, even if the potential energy surfaces are close in energy.

Based on the above discussion, we only need to focus on the most favored ^4^ **A** pathway when discussing the electronic states along the oxygen-activation process. The generation of ^4^ **IM2A**_Mn_ via ^4^ **TS1A**_Mn_ shares the same electron transfer route as ^5^ **TS1A**_Fe_, where one electron is transferred from the 3d orbital of the metallic center to the π* orbital of ^3^O_2_, and the lone pair of electrons of O^d^ attacks the C^2^ atom to form ^4^ **IM2A**_Mn_, as depicted in Figure 4c. The Mn atom in ^4^ **IM2A**_Mn_ has an SDD population number of 4.03 e, behaving as a high-spin d^4^ configuration of Mn^III^, which is consistent with the one-electron transfer mechanism. However, while the Fe-catalyzed ^5^ **TS2A**_Fe_ leads to radical decarboxylation (Figure 3c), no SDD distribution is observed on the C^1^–C^2^ bond in either ^4^ **TS2A**_Mn_ or ^6^ **TS2**_Mn_, which indicates that the C^1^–C^2^ bond undergoes heterolysis during the decarboxylation process. The electron transfer route should therefore be described as shown in Figure 4c, where one electron is transferred from the 3d orbital of the Mn atom to the O–O π* orbital, and the Mn atom receives another pair of electrons from the carboxyl and is reduced to Mn^II^.

After the formation of the peroxide intermediate ^4^ **IM3**_Mn_, the Mn-catalyzed peroxide cleavage shares a similar heterolysis mechanism with the Fe-catalyzed reaction, as shown in Figure 4b, and the SDD isosurface is only observed on the Mn atom in both ^4^ **IM3**_Mn_ and ^4^ **TS3**_Mn_. However, the Mn atom in ^4^ **IM4**_Mn_ has an SDD population number of 4.01 e, which corresponds to the high-spin d^4^ configuration of Mn^III^, while another unpaired electron is mainly located on the O atom, with an SDD population number of −0.95 e. This demonstrates that the electronic state of ^4^ **IM4**_Mn_ (or ^4^ **Cpd I**_Mn_) should be described as a Mn^III^–oxyl complex instead of a Mn^IV^–oxo complex, which is also the case for the sextet state (^6^ **IM4**_Mn_).

#### 3.2.2 CoZn_4_(prv)_4_(btdd)_3_

In biochemical systems, Mn and Fe are common metallic centers used for oxygen activation, while Co is rarely reported to participate in redox reactions, although three oxidation states (+1, + 2, and +3) are biochemically available in vitamin B_12_-dependent enzymes to mediate radical reactions. One possible reason is that Co is not as abundant as Mn or Fe in nature; however, this phenomenon may also be related to the reactivity of Co-containing complexes. Considering that Co^II^ has an ionic radius similar to that of Mn and Fe, we therefore replaced Fe^II^ with Co^II^ in the resting state, which has one more electron and gives CoZn_4_(prv)_4_(btdd)_3_ as a model to investigate the oxygen-activation reactivity of the nonheme Co^II^ center.

The Gibbs free energy diagram for the Co-catalyzed pathway is displayed in Figure 5a. For the heme-like Co center in vitamin B_12_, the macrocyclic ligand usually generates a strong ligand field and prefers a low-spin metallic center.^54,55^ Nonheme Co-containing enzymes have not yet been discovered in nature, and the preference for spin states should follow that of the Fe-containing systems to be in the high-spin resting state. This is also verified by the DFT results, which also showed that Co^II^ in the resting state has a high-spin d^7^-configuration. The noncovalent adsorption structure of ^3^O_2_ will then take a quartet state (*S* = 3/2), as shown in Figure 5a, and this further explains that covalent adsorption at the doublet state (*S* = 1/2) will have higher energy (6.6 kcal·mol^–1^), in the form of a Co^III^–superoxo complex after SET from Co^II^ to ^3^O_2_.

**Figure 5:**
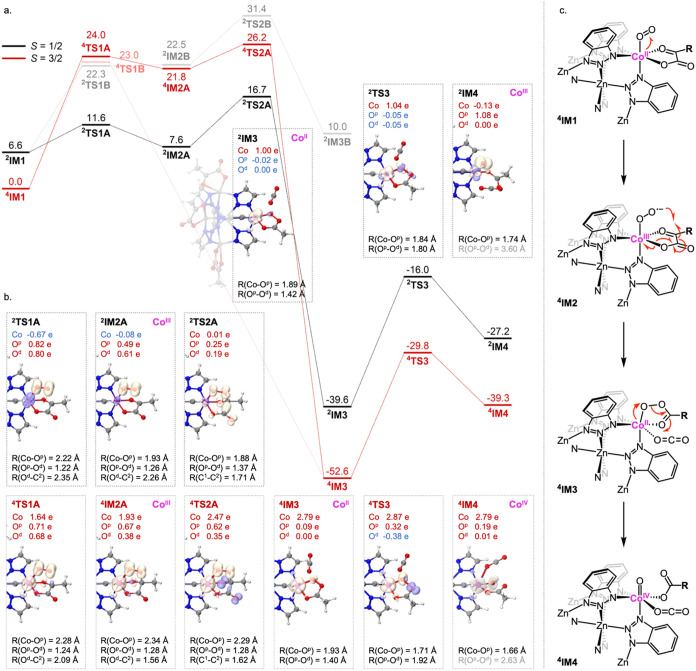
(a) Multispin-state Gibbs free energy diagram of oxygen activation by CoZn_4_(prv)_4_(btdd)_3_ (energy in kcal·mol^–1^ relative to ^4^ **IM1**). (b) Structure of the active site and spin density difference. (c) Electron transfer route of the favored reaction pathway.

The reaction pathway on the quartet state has Gibbs free energy barriers of 24.0 kcal·mol^–1^ at ^4^ **TS1A**_Co_ and 23.0 kcal·mol^–1^ at ^4^ **TS1B**_Co_, which are the rate-determining steps of the quartet pathway. There are two distinct reaction mechanisms: for ^4^A, the distance between the O^d^–C^2^ gradually shortens, followed by the formation of a new O^d^–C^2^ bond via the transition-state ^4^ **TS1A**_Co_ to yield intermediate ^4^ **IM2A**_Co_; for ^4^B, the nucleophilic attack of O^d^ on the C^2^ atom occurs concurrently with the elongation of the C^1^–C^2^ bond, triggering decarboxylation and directly forming intermediate ^4^IM3_Co_. In the doublet state, compared to ^4^ **IM1**_Co_, the Co–O^p^ and O^d^–C^2^ distances are shortened to 2.22 and 2.95 Å, respectively, suggesting an enhanced propensity for the nucleophilic attack. Our subsequent computational results further confirm this observation. Two reaction pathways emerge depending on whether antiferromagnetic coupling occurs between Co and O_2_: pathway **A** (with coupling) and pathway **B** (without coupling). The spin density difference population numbers are shown in Table S1. The potential energy surface clearly shows that all transition states and intermediates in ^2^B are higher in energy than those in ^2^A; additionally, the doublet pathway has a much lower barrier of 5.0 kcal·mol^–1^ at ^2^ **TS1A**_Co_. The O–O cleavage step (**TS3**_Co_) on either the doublet or quartet state has a similar barrier of 23.6 kcal·mol^–1^ or 23.8 kcal·mol^–1^, which indicates that the O–O cleavage transition state is not influenced by the spin state of the Co center and is a nonradical bond cleavage. This is also verified by the SDD distribution in Figure 5b, as no radical feature is observed for ^2^ **TS3**_Co_ and ^4^ **TS3**_Co_, which is consistent with the previously discussed Fe- or Mn-catalyzed pathways. ^2^ **TS3**_Co_ also acts as the rate-determining step in the doublet pathway. Comparing the barriers on all three metallic centers discussed in this work, we should consider that the Co center has the least reactivity toward oxygen activation.

The reactive oxygen species **IM4**_Co_ generated by CoZn_4_(prv)_4_(btdd)_3_ has the high-spin-state ^4^ **IM4**_Co_, which is 12.1 kcal·mol^–1^ lower in energy compared with the low-spin state ^2^ **IM4**_Co_. As revealed by the SDD population numbers, the unpaired electron of the low-spin state is mainly localized on the oxygen atom (1.06 e), corresponding to the electronic state of the Co^III^–oxyl complex. The Co–oxygen complex is less commonly reported compared with other early transition metals; however, the Co^III^–oxyl electronic state has already been verified in experiments with macrocyclic ligands such as porphyrin, corrin, or their derivatives. The comparison between the heme-type iron center and the nonheme-type iron centers shows that, at the nonheme site, a flexible coordinating environment with a weaker ligand field tends to yield a high-spin state at the metal center. Based on our calculations on CoZn_4_(prv)_4_(btdd)_3_, the conclusion can be extended to Co-centered systems where the high-spin state is the ground state for the Co–oxygen complexes and thus gives the Co^IV^–oxo complex electronic state, with the SDD population number reaching 2.79 e on the Co atom.

Although oxygen activation by CoZn_4_(prv)_4_(btdd)_3_ has a higher barrier and can be difficult to realize at room temperature, there are many studies that indicate that such a process is available with light excitation. A typical example is the photoinduced degradation of EDTACo^II^ in the presence of peroxymonosulfate (PMS), a systematically controllable model system for the simultaneous removal of transition metals and organic ligands via advanced oxidation processes (AOPs), which is a hot topic in environmental chemistry. Although the EDTACo^II^/PMS/light system exhibited high efficiency, none of the reactions were observed to degrade without light excitation, providing indirect evidence that oxygen activation by the Co center is not available via thermal activation in a nonheme-type ligand environment, even if a more reactive oxidant, such as PMS, is provided. On the basis of our results for the CoZn_4_(prv)_4_(btdd)_3_ system, we can also predict the existence of a Co^IV^–oxo intermediate in the light-induced oxidative degradation of EDTACo^II^. Mayer bond order analysis^48^ of representative transition states (Table S4) further shows different extents of M–O^p^ bond formation and O^p^–O^d^ bond activation in the Fe, Mn, and Co systems. From ^4^ **TS1**A_Co_ to ^4^ **TS3**_Co_, the Co–O^p^ MBO increases from 0.30 to 1.04, whereas the O^p^–O^d^ MBO decreases from 1.18 to 0.43 (Table S4), indicating progressive Co–O bond formation and O–O bond activation. Together with the Fe and Mn results, these MBO changes are consistent with the metal-dependent transition-state characteristics and mechanistic differences.

### 3.3 Structure and Reactivity of TM–Oxo/Oxyl Complexes

In low-spin cases, the TM–oxo complex is not available for d^6^-configuration, or further, i.e., the M–O π bond cannot be formed, which is known as the “oxo-wall” phenomenon.^56–58^ Considering MOL_5_-type TM–oxo complexes with ideal *C*_4*v*_ symmetry as a typical example, according to ligand field theory, orbital interactions between d orbitals and 2p orbital of the oxo ligand are displayed in Figure 6a. The M–O σ bond is formed between the d_*z*2_ and p_*z*_ orbitals, generating a low-lying bonding orbital, usually noted as σ_*z* ^2^_ orbital (or simply σ_*z*_) and a corresponding antibonding σ_*z* ^2^_^*^ orbital (or simply σ_z_^*^). If formed, two M–O π bonds are perpendicular to each other in the *xz* and *yz* planes, composed of d_*xz*_ and 2p_*x*_ orbitals or d_*yz*_ and 2p_*y*_ orbitals, respectively, which correspond to two pairs of degenerated π and π* orbitals. In addition, the d_*xy*_ and d_*x*2–*y*2_ orbitals are predominantly nonbonding with respect to the axial oxygen. In oxo complexes, the σ_*z* ^2^_, π_*xz*_, and π_*yz*_ orbitals are fully occupied by the 2p electrons of the oxo ligand, and 3d electrons of the transition-metal center fill the higher energy levels, as marked in the box in Figure 6a. For low-spin cases, the M–O triple bond can be formed by the d^0^- to d^2^-configuration with the nonbonding d_*xy*_ becoming populated. Since the d^3^-configuration starts to occupy the M–O π_xz_^*^ and π_yz_^*^ orbitals and M–O π bond is weakened. For the d^6^-configuration and further, the M–O π bond is no longer formed with both π_xz_^*^ and π_yz_^*^ orbitals doubly occupied.

**Figure 6:**
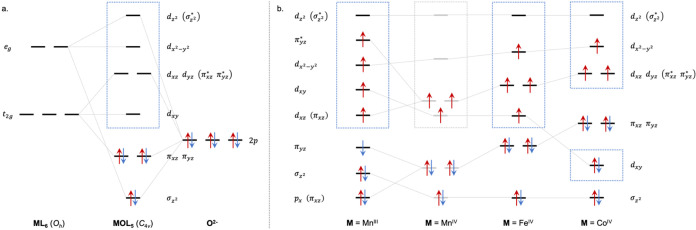
(a) General orbital interaction scheme in the *C*_4*v*_ ligand field for MOL_5_-type the oxo complex and (b) orbital energy level crossing in the high-spin Mn^III^, Mn^IV^, Fe^IV^, and Co^IV^–oxo/–oxyl complexes.

The most studied and applied TM–oxo complex is the Fe^IV^–oxo complex (d^4^-configuration), which has been proven to be crucial for the redox reactivity of many Fe-containing enzymes and related catalysts. Both triplet and quintet states are available for the Fe^IV^–oxo complex. With a strong field macrocyclic ligand like porphyrin, **Cpd I**_Fe,P450_ has a triplet Fe^IV^ center with a doubly occupied d_*xy*_ orbital, and π_xz_^*^ and singly occupied π_yz_^*^ orbitals. This has been extensively discussed in P450-related catalytic systems. However, for the nonheme Fe^IV^–oxo complex, a high-spin quintet state is usually observed as the ground state, and the electron configuration therefore follows, as shown in Figure 6b. This has been demonstrated in the Fe^II^/2-OGDC-related catalytic systems. Here, the unrestricted natural orbitals (UNOs) of ^5^ **IM4**_Fe_ in Figure 3a are sorted according to the natural orbital occupation number (NOON) and are listed in Figure S3b. It can be seen that the d_*xy*_ orbital has NOON of 1.9, which is close to that of the doubly occupied. Mixed with 3d and 2p orbitals, HOMO and HOMO-1 show π symmetry and have nearly degenerated NOON of 1.0 and 1.0, corresponding to the π_xz_^*^ and π_yz_^*^ levels, respectively. This further demonstrates that the electronic structure of ^5^ **IM4**_Fe_ fits well with the bonding scheme shown in Figure 6b, sharing the same form of the Fe^IV^–oxo complex as **Cpd I**_Fe,P450_.

Several studies have demonstrated the stability of Mn^V^–oxo complexes (d^2^-configuration),^59–61^ and according to the “oxo-wall” phenomenon, the stability of the Mn^IV^–oxo complex (d^3^-configuration) should be between those of Mn^V^–oxo and Fe^IV^–oxo complexes. However, for ^4^ **IM4**_Mn_ in Figure 4a, the resonance structure of the Mn^III^-oxyl complex is observed instead of the Mn^IV^–oxo complex, as discussed in previous sections. The UNOs of ^4^ **IM4**_Mn_ are sorted according to the NOON in Figure S3a, and the bonding scheme is summarized in Figure 6b. Compared with the standard MOL_5_ model (or Fe^IV^–oxo complex), the antiferromagnetic ground state is observed for ^4^ **IM4**_Mn_, and π_*xz*_ and π_*yz*_ orbitals are asymmetrically occupied with three electrons, where, according to the Jahn–Teller effect, degenerated orbitals are further split to decrease the system energy, as indicated by the red connecting lines. In parallel, the π_xz_^*^ and π_yz_^*^ orbitals are no longer degenerated. In this case, the 2p_*x*/*y*_ orbitals of the O atom and 3d_*xz*/*yz*_ orbitals of Mn are not coupled and behave as localized orbitals.

According to the “oxo-wall”, for low-spin cases, Co^III^ with a d^6^-configuration cannot form a Co^III,LS^–oxo complex, as both the π_xz_^*^ and π_yz_^*^ orbitals are fully occupied. Instead, the Co^III,LS^–oxyl complex is the preferred electronic state and can be generated by providing peroxides as oxidants, which has been proven by both experimental and theoretical studies in vitamin B_12_-related systems.^62^ The corrin ligand in vitamin B_12_ has limited space for the Co^III^ center, so that stronger metal–ligand interactions usually lead to a low-spin electron configuration. Li et al.^63^ and Akogun et al.^64^ sucessfully detected the Co^III,LS^–oxyl complex by EPR. As shown in Figure 5b, in the doublet state, ^2^ **IM4**_Co_ is observed to be a Co^III,LS^–oxyl complex, as the SDD isosurface is only distributed on the 2p orbital of the O atom, while the rest of the distribution on the Co center presents a higher angular momentum component and should be viewed as numerical results brought by the extended basis applied in calculations. However, the Co^III,HS^–oxyl complex is unstable if formed, as the singly occupied σ_*z* ^2^_^*^ orbital leads to a weakened Co–O σ bond. Meanwhile, the Co^IV,HS^–oxo complex is possible as the π_xz_^*^ and π_yz_^*^ orbitals are both singly occupied. For ^4^ **IM4**_Co_ in Figure 5b, UNOs present a similar bonding scheme to that of the Fe^IV^–oxo complex with one more electron populating on the d_*xy*_ orbital (Figure 6b). This is an important difference between Co and Fe. By adjusting the coordinating environment, both the low- and high-spin states of Fe prefer the Fe^IV^–oxo complex instead of the Fe^III^–oxyl complex. However, with one more electron, Co shows different preferences and so distinct reactivities on different spin states, indicating that potential efforts can be made to explore the multistate redox reactivity of the synthesized Co-containing catalysts.

The difference in the bonding schemes of Mn, Fe, and Co can be understood from ligand field theory. Considering the high-spin TM–oxo complex form of the three metallic centers, the ionic radius decreases from Mn, Fe to Co, leading to stronger metal–ligand interactions, and a larger ligand field splitting energy, which makes the energy of the d_*xy*_ orbital comparatively lower, yet *d*_*x* ^2^–*y* ^2^_, π_xz_^*^, and π_yz_^*^ orbitals become comparatively higher, as indicated in Figure 6b.

Assuming that the Mn^IV^–oxo complex exists for MnZn_4_(prv)_4_(btdd)_3_, the smaller d-π energy gap brought by the lower *d*_*x* ^2^–*y* ^2^_, π_xz_^*^, and π_yz_^*^ orbitals compared with Fe^IV^ enhances the ligand-to-metal electron transfer. The high redox potential of the Mn^IV^ center may further help this, which finally leads to π → d SET and gives the Mn^III^–oxyl complex as the ground electronic state. Moreover, since the loss of one electron on the π orbitals results in one unpaired electron, the unsymmetrically occupied π_*xz*_ and π_*yz*_ orbitals lead to further splitting of the π orbitals and π* orbitals, as pointed out by the Jahn–Teller effect. This explains the unique bonding scheme of the Mn^III^–oxyl complex displayed in Figure 6b. Another minor difference for the Co^III,HS^–oxo complex is that the d_*xy*_ orbital is lower than the π orbitals due to the larger ligand field splitting energy. For the high-spin d^5^-configuration, the quintet state is usually available for a smaller splitting energy.

## 4 Conclusion

The application of triplet oxygen as an oxidant in catalysis is challenging due to its low reactivity toward most closed-shell organic substrates. Biological solutions have been successful in constructing different transition-metal-catalyzed oxygen-activation pathways, and biomimetic materials have provided much insight into biological processes. On the basis of the recently reported structure of FeZn_4_(prv)_4_(btdd)_3_ MOFs, which reconstructs the active site of Fe^II^/2-OGDC enzymes, we investigated the oxygen activation reactivity of FeZn_4_(prv)_4_(btdd)_3_ using DFT calculations and explored the possibility of replacing Fe^II^ with Mn^II^ or Co^II^.

Computational results showed that FeZn_4_(prv)_4_(btdd)_3_ shares the same oxygen-activation mechanism as Fe^II^/2-OGDC enzymes, applying a quintet state Fe^IV^–oxo complex (^5^ **IM4**_Fe_, or **Cpd I**_Fe_) as a reactive oxygen species. Similarly, a Co^IV^–oxo complex is also formed in the high-spin quartet state of ^4^ **IM4**_Co_ (or **Cpd I**_Co_), with one more electron occupying the d_*x*2‑*y*2_ orbital compared with the Fe^IV^–oxo complex. However, for ^4^ **IM4**_Mn_ (or **Cpd I**_Mn_), the preferred electronic state is the Mn^III^-oxyl complex instead of the Mn^IV^–oxo complex due to the Jahn–Teller effect caused by ligand-to-metal single-electron transfer. The oxygen-activation mechanism of TMZn_4_(prv)_4_(btdd)_3_ (TM = Mn, Fe, or Co) was also proven to be the same as that of Fe^II^/2-OGDC enzymes. Reaction energy profiles obtained by DFT calculations also suggested that the generation of **Cpd I**_Mn_ and **Cpd I**_Fe_ species is thermodynamically favored at room temperature, while the generation of **Cpd I**_Co_ has a higher Gibbs free energy barrier and is therefore not available. This indicates that replacing Fe with Mn may result in a similar catalytic performance, while Co may not.

The present results show that the different reactivities of the Fe, Mn, and Co systems are not determined by barrier heights alone but arise from the combined effects of spin-state accessibility, metal–oxygen bonding, and O–O activation ability. Fe and Co favor high-spin TM^IV^–oxo species, whereas Mn stabilizes a high-spin Mn^III^–oxyl species, reflecting clear metal-dependent differences in the electronic structure. Meanwhile, the lower oxygen-activation barriers in the Fe and Mn systems than in the Co analogue indicate that an effective Fe^II^/2-OGDC-like catalyst should provide both accessible reactive spin states and a favorable balance between metal–oxygen bond formation and O–O bond activation. This mechanistic picture offers useful design insights for developing MOF-based biomimetic oxidation catalysts with alternative transition metals.

## Supplementary Material





## Notes

LOBA assignments for all atoms; SDD population numbers for all atoms (ZIP)
Electronic configurations of Fe^II^–O_2_, electron transfer routes of ^3^TS1A_Fe_ and ^5^TS1A_Fe_; unrestricted natural orbitals (UNO) of ^4^Cpd I_Mn_, ^4^Cpd I_Fe_, and ^4^Cpd I_Co_; IRC curves for ^5^TS1A_Fe_, ^5^TS3_Fe_, and ^4^TS3_Mn_; SDD population numbers for metals, O^p^, and O^d^; spin contamination values (<S^2^>); LOBA assignments for metals; Mayer bond order; imaginary frequency of the transition states (PDF)
