# Supplementary material for: Elucidating the Oxygen-Activation Mechanism in Nonheme MnII-, FeII-, or CoII-Containing MOFs Mimicking FeII/2-Oxoglutarate-Dependent Complexes
Source: Inorg Chem. 2026 May 21;65(22):12206–19. doi: 10.1021/acs.inorgchem.6c00307 (PMC13250979; doi:10.1021/acs.inorgchem.6c00307)
Supplement: Supplementary file 2 [file ic6c00307_si_002.pdf]

Supporting Information

Elucidating Oxygen Activation Mechanism by Non-Heme Mn^{II}, Fe^{II}, or Co^{II}-Containing MOFs Mimicking Fe^{II}/2-Oxoglutarate-Dependent Complex

Ziyue Huang,[1] Yingqi Li[1], Xiaotian Zhang,[1] Xi Chen[1], Jiawei Xu[2,*] and Haiyan Wei[1,*]

1. Jiangsu Key Laboratory of Biofunctional Materials, School of Chemistry and Materials Science, Ministry-of-Education Key Laboratory of Numerical Simulation of Large-Scale Complex Systems, Nanjing Normal University, Nanjing 210023, Jiangsu, China.

2. Physical and Theoretical Chemistry Laboratory, Department of Chemistry, University of Oxford, Oxford OX1 3QZ, United Kingdom.

* Corresponding authors: jiawei.xu@chem.ox.ac.uk (J. Xu) and weihaiyan@njnu.edu.cn (H. Wei)

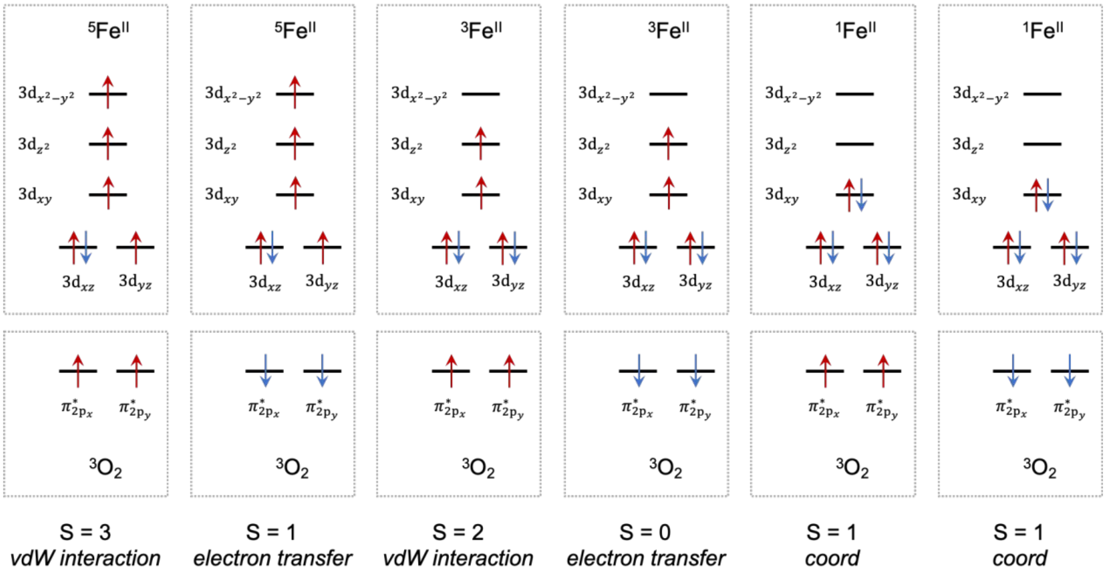

Figure S1. All possible spin-state combinations of Fe^{II} and $^3O_2$.

There are two dominant electronic configurations: Fe^{II}-O_2, formed by direct coordination between Fe^{II} and $^3O_2$, where $^3O_2$ donates LP to $3d_{z2}$ orbital of Fe^{II}; or Fe^{III}-superoxo, formed by Fe^{II}-to-$^3O_2$ single electron transfer. Structure with the lowest energy holds $S = 3$ and forms vdW interaction distance between spin-paralleled Fe and O_2, as has been discussed in main text. As for structures with Fe-O bond, the minimum energy structure comes from spin-opposite combination of $^5Fe^{II}$ and $^3O_2$, which results in $S = 1$ state and opposite spin on Fe and O_2 allows single electron transfer from $3d_{xz}$ to π^*. This will generate an unstable electronic configuration on Fe^{III} centre and an MECP is required to connect $S = 1$ and $S = 2$ states (see **Figure 3** and related discussion in main text). All other possible combinations start from medium- or low-spin Fe^{II} centre, while in non-heme-type coordination environment, medium- and low-spin Fe^{II} are unavailable in energy.

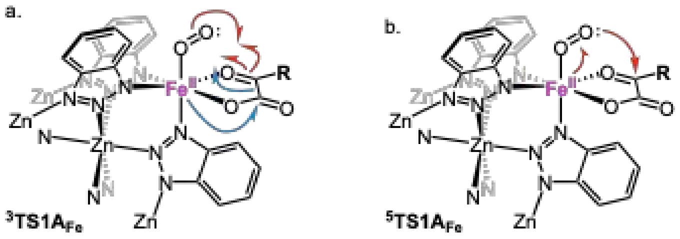

Figure S2. The electron transfer route in **(a)** ^{3}**TS1A**$_{Fe}$ and **(b)** ^{5}**TS1A**$_{Fe}$.

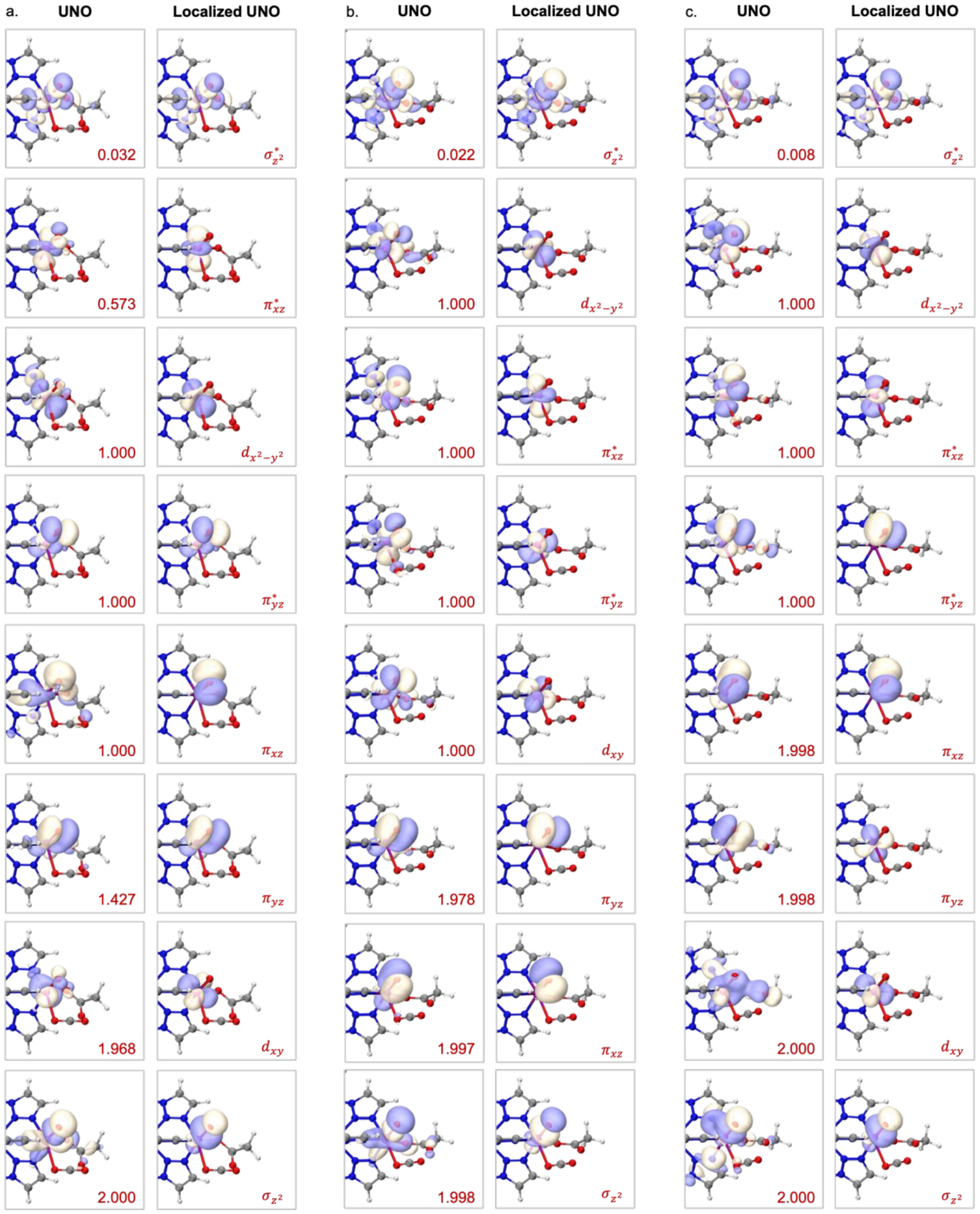

Figure S3. Unrestricted natural orbital (UNO) and localized UNO for **(a)** ^{4}**Cpd I**$_{\text{Mn}}$, **(b)** ^{4}**Cpd I**$_{\text{Fe}}$, and **(c)** ^{4}**Cpd I**$_{\text{Co}}$, sorted by natural orbital occupation number (NOON) as marked.

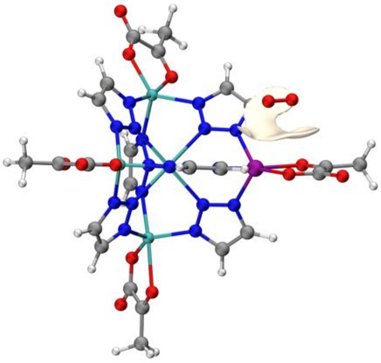

Figure S4. IGMH isosurface of ^{7}**IM1**$_{Fe}$.

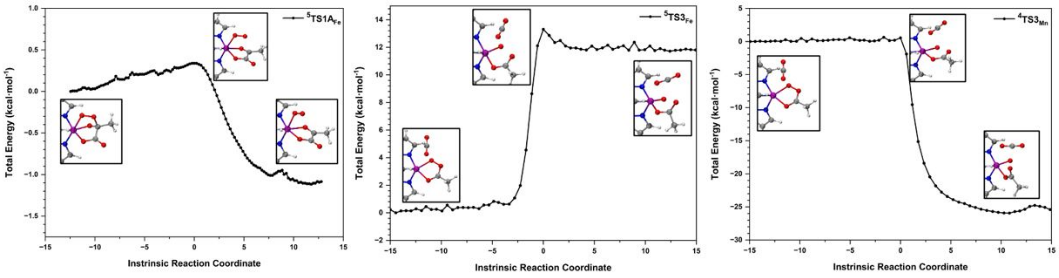

Figure S5. Intrinsic Reaction Coordinate (IRC) curve for ^{5}TS1A$_{Fe}$, ^{5}TS3$_{Fe}$ and ^{4}TS3$_{Mn}$.

Table S1. Selected spin density difference (SDD) population numbers of the key atoms in representative optimized structures of the Fe, Mn, and Co systems. Only the values directly discussed in the main text are listed here. the complete atomic SDD population outputs are provided in the accompanying .pop files in the SI zip package.

	TM	O^p	O^d
^{2}IM1$_{Co}$	-0.80	0.92	0.85
^{2}TS1A$_{Co}$	-0.67	0.82	0.80
^{2}TS1B$_{Co}$	2.78	-0.63	-0.51
^{2}IM2A$_{Co}$	-0.08	0.61	0.49
^{2}IM2B$_{Co}$	2.78	-0.63	-0.42
^{2}TS2A$_{Co}$	0.01	0.25	0.19
^{2}TS2B$_{Co}$	2.53	-0.62	-0.39
^{2}IM3A$_{Co}$	1.00	-0.02	-0.00
^{2}IM3B$_{Co}$	2.79	0.09	-0.00
^{2}TS3A$_{Co}$	1.04	-0.05	-0.05
^{2}IM4A$_{Co}$	-0.12	1.08	0.00
^{4}IM1$_{Co}$	2.82	0.01	0.00
^{4}TS1A$_{Co}$	1.64	0.71	0.68
^{4}TS1B$_{Co}$	2.76	0.42	0.27
^{4}IM2A$_{Co}$	1.93	0.67	0.38
^{4}TS2A$_{Co}$	2.47	0.62	0.35

^{4}IM3$_{Co}$	2.79	0.09	-0.00
^{4}TS3$_{Co}$	2.87	0.32	-0.38
^{4}IM4$_{Co}$	2.79	0.09	-0.00
^{3}IM2A$_{Fe}$	2.99	-0.58	-0.36
^{3}TS1A$_{Fe}$	3.81	-0.64	-0.46
^{3}IM3$_{Fe}$	2.01	0.03	-0.01
^{3}TS2A$_{Fe}$	3.02	-0.40	-0.34
^{3}IM4$_{Fe}$	1.28	0.81	0.00
^{3}TS3$_{Fe}$	2.22	-0.01	-0.17
^{5}IM2A$_{Fe}$	4.32	-0.51	-0.39
^{5}TS1A$_{Fe}$	4.20	-0.12	-0.44
^{5}IM3$_{Fe}$	3.85	0.07	0.01
^{5}TS2A$_{Fe}$	4.30	-0.13	-0.27
^{5}IM4$_{Fe}$	3.17	0.69	0.02
^{5}TS3$_{Fe}$	4.04	0.07	-0.28
^{2}IM1$_{Mn}$	2.30	-0.51	-0.70
^{2}IM2$_{Mn}$	2.09	-0.55	-0.43
^{2}TS2$_{Mn}$	2.14	-0.34	-0.31
^{2}IM3$_{Mn}$	3.07	-0.01	-0.01
^{4}IM1$_{Mn}$	4.87	-0.94	-1.00
^{4}TS1A$_{Mn}$	4.04	-0.50	-0.54
^{4}TS1B$_{Mn}$	2.26	0.45	0.42
^{4}IM2A$_{Mn}$	4.04	-0.60	-0.36
^{4}IM2B$_{Mn}$	3.05	0.03	0.05
^{4}TS2A$_{Mn}$	4.04	-0.33	-0.31
^{4}TS2B$_{Mn}$	2.91	0.05	0.06
^{4}IM3$_{Mn}$	3.07	-0.01	-0.01
^{4}TS3$_{Mn}$	3.15	-0.04	-0.07
^{4}IM4$_{Mn}$	4.01	-0.95	0.01
^{6}IM1$_{Mn}$	4.81	0.03	0.10
^{6}TS1$_{Mn}$	4.10	0.40	0.53
^{6}IM2$_{Mn}$	4.02	0.65	0.37
^{6}TS2$_{Mn}$	4.02	0.30	0.32
^{6}IM3$_{Mn}$	4.90	0.03	0.01
^{6}TS3$_{Mn}$	4.51	0.17	0.30
^{6}IM4$_{Mn}$	3.96	0.98	0.03

Table S2. Spin contamination values ($\langle S^2 \rangle$) for the optimized structures.

	IM1	TS1A	TS1B	IM2A	IM2B	TS2A	TS2B	IM3	IM3B	TS3	IM4
^{3}Fe	3.97	3.70	2.32	3.06	2.02	3.05	2.02	2.01		2.16	2.03
^{5}Fe	6.88	6.95	6.10	6.99	6.06	6.83	6.07	6.01		6.38	6.06
^{7}Fe	12.02										
^{2}Mn	1.85			1.73				2.78			
^{4}Mn	5.69	4.79	3.78	4.80	3.83	4.76	3.81	3.77		3.82	4.64

^{6}Mn	9.59	8.84		8.81		8.81		8.75		8.91	8.79
^{2}Co	1.48	1.31	2.68	0.77	2.70	0.76	2.35	0.94	2.76	1.43	1.15
^{4}Co	4.75	3.81	4.61	3.81		4.28		3.76		3.87	3.78

The spin expectation values were further examined to evaluate possible spin contamination in the unrestricted Kohn-Sham solutions. Several early O_2-bound and antiferromagnetically coupled intermediates show noticeable deviations from the ideal $\langle S^2 \rangle$ values, especially for the triplet Fe, quartet Mn, and doublet Co surfaces. These deviations are expected for broken-symmetry descriptions of open-shell metal-dioxygen species and indicate that the corresponding spin-state gaps and barriers should be interpreted semi-quantitatively. Importantly, the largest deviations are mainly found for early O_2-adducts or less favorable low-spin surfaces, whereas the spin contamination of key high-valent oxidizing intermediates are acceptable. Therefore, the spin contamination does not alter the qualitative mechanistic conclusions, namely the formation of high-spin Fe^{IV}-oxo and Co^{IV}-oxo species and the preferential Mn^{III}-oxyl character in the Mn analogue, although it introduces uncertainty into the absolute energies of strongly broken-symmetry states.

Table S3. LOBA assignments obtained with different localization thresholds for optimized structures in the Fe, Mn, and Co systems. Only the key assignments relevant to the mechanistic discussion are tabulated here. The complete LOBA outputs are provided in the .ox files in the SI zip package.

	LOBA-30	LOBA-40	LOBA-50	LOBA-60	LOBA-70	mLOBA
^{3}IM2A$_{Fe}$	3	3	3	3	3	3
^{5}IM2A$_{Fe}$	3	3	3	3	3	3
^{3}IM3$_{Fe}$	2	2	2	2	2	2
^{5}IM3$_{Fe}$	2	2	2	2	2	2
^{3}IM4$_{Fe}$	0	3	4	4	4	4
^{5}IM4$_{Fe}$	1	2	4	4	4	4
^{3}TS1A$_{Fe}$	2	2	2	2	2	2
^{5}TS1A$_{Fe}$	3	3	3	3	3	3
^{3}TS2A$_{Fe}$	3	3	3	3	3	3
^{5}TS2A$_{Fe}$	3	3	3	3	3	3
^{3}TS3$_{Fe}$	2	2	2	2	3	2
^{5}TS3$_{Fe}$	2	2	3	3	3	3
^{2}IM1$_{Mn}$	3	3	3	3	3	3
^{4}IM1$_{Mn}$	2	2	2	2	2	2
^{6}IM1$_{Mn}$	2	2	2	2	2	2
^{2}IM2$_{Mn}$	3	3	3	3	3	3
^{6}IM2$_{Mn}$	3	3	3	3	3	3
^{4}IM2A$_{Mn}$	3	3	3	3	3	3
^{4}IM2B$_{Mn}$	4	4	4	4	4	4
^{2}IM3$_{Mn}$	2	2	2	2	2	2
^{4}IM3$_{Mn}$	2	2	2	2	2	2
^{6}IM3$_{Mn}$	2	2	2	2	2	2
^{4}IM4$_{Mn}$	2	3	3	3	3	3
^{6}IM4$_{Mn}$	3	3	3	3	3	3
^{6}TS1$_{Mn}$	3	3	3	3	3	3
^{4}TS1A$_{Mn}$	3	3	3	3	3	3
^{4}TS1B$_{Mn}$	3	3	3	3	3	3

$^{2}TS2_{Mn}$	3	3	3	3	3	3
$^{6}TS2_{Mn}$	3	3	3	3	3	3
$^{4}TS2A_{Mn}$	3	3	3	3	3	3
$^{4}TS2B_{Mn}$	4	4	4	4	4	4
$^{4}TS3_{Mn}$	2	2	2	2	2	2
$^{6}TS3_{Mn}$	2	2	2	2	3	2
$^{2}IM1_{Co}$	2	2	2	2	2	2
$^{2}IM2A_{Co}$	3	3	3	3	3	3
$^{2}IM2B_{Co}$	2	2	2	2	2	2
$^{2}IM3A_{Co}$	2	2	2	2	2	2
$^{2}IM3B_{Co}$	2	2	2	2	2	2
$^{2}IM4A_{Co}$	2	3	3	3	3	3
$^{2}TS1A_{Co}$	2	2	2	2	3	2
$^{2}TS1B_{Co}$	2	2	2	2	2	2
$^{2}TS2A_{Co}$	3	3	3	3	3	3
$^{2}TS2B_{Co}$	2	2	2	2	2	2
$^{2}TS3A_{Co}$	2	2	2	2	2	2
$^{4}IM1_{Co}$	2	2	2	2	2	2
$^{4}IM2A_{Co}$	3	3	3	3	3	3
$^{4}IM3_{Co}$	2	2	2	2	2	2
$^{4}IM4_{Co}$	2	3	3	3	3	3
$^{4}TS1A_{Co}$	3	3	3	3	3	3
$^{4}TS1B_{Co}$	2	2	2	2	2	2
$^{4}TS2A_{Co}$	2	2	2	2	2	2
$^{4}TS3_{Co}$	2	2	2	3	3	2

Table S4. Mayer bond order analysis for selected representative transition states of the Fe, Mn, and Co systems, showing the $M\text{-}O^{p}$ and $O^{p}\text{-}O^{d}$ bond orders.

	$M\text{-}O^{p}$	$O^{p}\text{-}O^{d}$
$^{3}TS1A_{Fe}$	0.25	1.13
$^{3}TS3_{Fe}$	1.03	0.57
$^{6}TS1_{Mn}$	0.44	1.14
$^{6}TS3_{Mn}$	0.79	0.42
$^{4}TS1A_{Co}$	0.30	1.18
$^{4}TS3_{Co}$	1.04	0.43

Table S5. Imaginary frequency for transition states of the Fe, Mn, and Co systems. Each optimized transition state has one and only one imaginary frequency, and the corresponding vibrational mode is associated with the intended bond-forming or bond-breaking coordinate.

	Imaginary Frequency (cm^{-1})
$^{3}TS1A_{Fe}$	351.2i
$^{3}TS1B_{Fe}$	59.1i
$^{5}TS1A_{Fe}$	68.2i
$^{5}TS1B_{Fe}$	147.4i

$^{3}TS2A_{Fe}$	359.7i
$^{3}TS2B_{Fe}$	179.5i
$^{5}TS2A_{Fe}$	528.0i
$^{5}TS2B_{Fe}$	358.9i
$^{3}TS3_{Fe}$	129.2i
$^{5}TS3_{Fe}$	501.4i
$^{4}TS1A_{Mn}$	146.8i
$^{4}TS1B_{Mn}$	319.7i
$^{6}TS1_{Mn}$	167.5i
$^{4}TS2A_{Mn}$	378.9i
$^{4}TS2B_{Mn}$	336.0i
$^{6}TS2_{Mn}$	408.5i
$4TS3_{Mn}$	563.2i
$^{6}TS3_{Mn}$	1004.2i
$^{2}TS1A_{Co}$	140.1i
$^{2}TS1B_{Co}$	212.9i
$^{4}TS1A_{Co}$	436.7i
$^{4}TS1B_{Co}$	381.1i
$^{2}TS2A_{Co}$	24.9i
$^{2}TS2B_{Co}$	756.6i
$^{4}TS2A_{Co}$	962.5i
$^{2}TS3_{Co}$	409.9i
$^{4}TS3_{Co}$	461.3i

Table S6. gCP estimates of BSSE contributions to selected Mn reaction barriers under dft/def2-TZVP level.

Barrier	gCP correction of IM/kcal·mol^{-1}	gCP correction of TS/kcal·mol^{-1}	BSSE contribution to barrier/kcal·mol^{-1}
$^{4}IM2A_{Mn}$-$^{4}TS2A_{Mn}$	46.6707	46.7822	0.1115
$^{6}IM2_{Mn}$-$^{6}TS2_{Mn}$	46.6626	46.7643	0.1017

For each individual barrier, the estimated gCP BSSE contribution is ca. 0.1 kcal·mol^{-1}: 0.1115 kcal·mol^{-1} for $^{4}IM2A_{Mn}$-$^{4}TS2A_{Mn}$ and 0.1017 kcal·mol^{-1} for $^{6}IM2_{Mn}$-$^{6}TS2_{Mn}$. More importantly, because the mechanistic discussion depends on the relative ordering of the competing quartet and sextet barriers, the relevant quantity is the differential BSSE contribution between these two barriers. This differential contribution is only 0.1115 - 0.1017 = 0.0098 kcal·mol^{-1}. Therefore, although BSSE contributes about 0.1 kcal·mol^{-1} to the absolute value of each tested barrier, its effect on the barrier trend and spin-state ordering is negligible.
